# Variability of sclerosis along the longitudinal hippocampal axis in epilepsy: A post mortem study

**DOI:** 10.1016/j.eplepsyres.2012.04.015

**Published:** 2012-11

**Authors:** Maria Thom, Ioannis Liagkouras, Lillian Martinian, Joan Liu, Claudia B. Catarino, Sanjay M. Sisodiya

**Affiliations:** a Department of Clinical and Experimental Epilepsy, UK; b Division of Neuropathology, Institute of Neurology and National Hospital for Neurology and Neurosurgery, Queen Square, London WC1N 3BG, UK; c Division of Clinical Neurology, Institute of Neurology and National Hospital for Neurology and Neurosurgery, Queen Square, London WC1N 3BG, UK; d National Society for Epilepsy, Chesham Lane, Chalfont St. Peter, Bucks SL9 0RJ, UK

**Keywords:** Hippocampal sclerosis, Quantitation, Calbindin, Neuropeptide Y, Calretinin

## Abstract

Detailed neuropathological studies of the extent of hippocampal sclerosis (HS) in epilepsy along the longitudinal axis of the hippocampus are lacking. Neuroimaging studies of patients with temporal lobe epilepsy support that sclerosis is not always localised. The extent of HS is of relevance to surgical planning and poor outcomes may relate to residual HS in the posterior remnant. In 10 post mortems from patients with long histories of drug refractory epilepsy and 3 controls we systematically sampled the left and right hippocampus at seven coronal anatomical levels along the body to the tail. We quantified neuronal densities in CA1 and CA4 subfields at each level using Cresyl Violet (CV), calretinin (CR), calbindin (CB) and Neuropeptide Y (NPY) immunohistochemistry. In the dentate gyrus we graded the extent of granule cell dispersion, patterns of CB expression, and synaptic reorganisation with CR and NPY at each level. We identified four patterns of HS based on patterns of pyramidal and interneuronal loss and dentate gyrus reorganisation between sides and levels as follows: (1) symmetrical HS with anterior–posterior (AP) gradient, (2) symmetrical HS without AP gradient, (3) asymmetrical HS with AP gradient and (4) asymmetrical cases without AP gradient. We confirmed in this series that HS can extend into the tail. The patterns of sclerosis (classical versus atypical or none) were consistent between all levels in less than a third of cases. In conclusion, this series highlights the variability of HS along the longitudinal axis. Further studies are required to identify factors that lead to focal versus diffuse HS.

## Introduction

Clinico-pathological studies spanning over 130 years have established a relationship between hippocampal sclerosis (HS) and epilepsy, in particular temporal lobe epilepsy (TLE) (Thom, 2009). In large post mortem epilepsy series, HS is bilateral in around half of cases (Meencke et al., 1996; Thom et al., 2011). In surgical series of patients with TLE, where HS is presumed to be a unilateral process, variable patterns of sclerosis, including classical and atypical types, have been noted (Blumcke et al., 2007; Bruton, 1988; de Lanerolle et al., 2003) in which specific subfield loss of neurones, usually based on a single coronal level of hippocampus, is identified. Following surgery for HS, approximately two thirds of patients will remain seizure-free in the first two to three years (Thom et al., 2010b) with figures of 57% for seizure-free outcomes at 5 years (de Tisi et al., 2011). The cause for surgical failure in most patients is unknown. It may relate to occult, bilateral, asymmetrical HS. There is conflicting evidence that atypical patterns of HS identified by neuropathology may predict a less favourable outcome (Blumcke et al., 2007; Bruton, 1988; Davies et al., 1996; de Lanerolle et al., 2003; Thom et al., 2010a; Van Paesschen et al., 1997). Moreover, pre-operative MRI is currently not able to categorise the subtype of hippocampal sclerosis (Eriksson et al., 2008; Howe et al., 2010). The extent of the hippocampal resection may also influence outcome (Wyler et al., 1995) and remnants of the hippocampus may be responsible for surgical failure. Several MRI studies have addressed the variation of hippocampal atrophy along its longitudinal axis (Bronen et al., 1995; Kuzniecky et al., 1996; Quigg et al., 1997; Woermann et al., 1998) but similar neuropathology studies are lacking. In addition to principal neuronal loss, there are specific patterns of interneuronal loss and reorganisation, arguably more relevant to the process of hippocampal epileptogenesis (Magloczky, 2010; Magloczky et al., 2000; Mathern et al., 1995; Wittner et al., 2002). In a previous study, we addressed the extent of bilateral hippocampal sclerosis and reorganisation in an epilepsy post mortem (PM) series with long durations of drug-resistant epilepsy (Thom et al., 2009). We now extend these studies to look at similar processes along the longitudinal hippocampal axis.

## Methods

### Case selection and sampling

Cases were selected from the neuropathology archives at the Institute of Neurology and the National Hospital for Neurology and Neurosurgery, London (UK). The study was approved by the Joint Research Ethics Committee of the Institute of Neurology and the National Hospital for Neurology and Neurosurgery. The post mortem (PM) cases were from patients with long histories of epilepsy. Era-appropriate consent for retention of tissue was granted by the next of kin. Clinical and pathology data are presented in Table 1. We included cases with varied original diagnosis based on examination of initial hippocampal samples, including classical, atypical HS, no HS and cases with evidence of bilateral neuronal loss. Two patients (cases EP254 and EP016) had undergone previous epilepsy surgery with partial right hippocampectomies (Table 1). Three PM control cases with no history of epilepsy and no neuropathological changes were included.

From all epilepsy and control PM cases, we sampled paired blocks of right and left mesial temporal lobe with hippocampus from the archival formalin-fixed tissue along the longitudinal hippocampal axis, at ten coronal levels in an anterior to posterior direction (a term we adopt throughout for rostro-caudal): Level 1 (nucleus accumbens), Level 2 (amygdala/anterior commisure), Level 3 (mammillary body), Level 4 (subthalamic nucleus), Level 5 (red nucleus), Level 6 (lateral geniculate nucleus), Level 7 (pulvinar-rostral), Level 8 (pulvinar-caudal), Level 9 (subsplenial) and Level 10 (hippocampal tail). We were not able to collect all levels from all cases and for the purposes of this study we focused on seven levels (4–10) where hippocampal subfields could be clearly defined. We excluded sections of hippocampal pes and head from further analysis. Levels 4–6 therefore represented anterior hippocampal body, levels 7–8 mid-body and levels 9–10, posterior hippocampus/tail. The formalin-fixation times of the epilepsy cases sampled varied from three to fourteen years and in the three controls was between nine and eleven years.

Twenty micron-thick sections were stained with Cresyl Violet (CV) for assessment and quantification of pyramidal cells. Seven micron-thick sections were used for immunohistochemical identification and quantitation of interneurones. Paraffin wax-embedded sections were dewaxed and rehydrated through graded alcohols and taken to water. Endogenous peroxidase was blocked with 3% hydrogen peroxide in deionized water for 15 min. Sections were microwaved in Vector Antigen Retrieval Solution (Vector: Burlington, CA, USA) then incubated with polyclonal markers against calbindin D-28K (CB) (1:10,000, Swant, Switzerland), calretinin (CR) 1:2000 (polyclonal, 1:2000; Sigma, Saint Louis, MO, USA) and Neuropeptide Y (NPY) (1:4000 Sigma). Antibodies were diluted in Dako ChemMate Diluent and labelling was detected with Dako Envision horse radish peroxidase (Dako, Glostrup, Denmark). Staining was visualised with Dako DAB+ Chromogen. Negative controls were treated identically except that the primary antibody was omitted. Between all steps sections were washed with PBS and 0.05% Tween 20. Parvalbumin, dynorphin, and NeuN staining were not consistent in long-term formalin fixed tissues, as we have previous shown (Liu et al., 2010), and were not included in the study. An additional 20 μm Cresyl Violet stained section was labelled with GFAP (polyclonal, 1:1000; DakoCytomation, Glostrup, Denmark) for the visualisation and confirmation of subfield gliosis, but these sections were not quantified. All sections from a single case with each antibody were stained during a single run.

### Qualitative analysis: reorganisation in dentate gyrus granule cell layer

**Figure 1:**
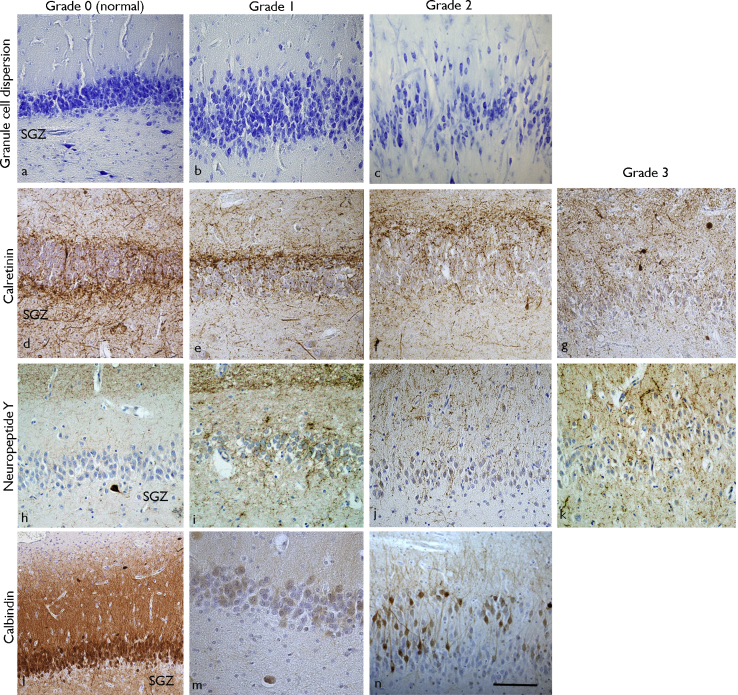
Grading of dentate gyrus reorganisation. (a–c) Granule cell dispersion (Cresyl Violet stain). Grade 0 (a) normal; Grade 1 (b) mild dispersion; Grade 2 (c) severe dispersion. (SGZ = sub granular zone). (d–g) Calretinin patterns in the dentate gyrus. (d) Normal (Grade 0) with dense bundles of fibres in the immediate inner molecular layer just above the granule cell layer and similar fibres in the sub granular zone (SGZ). (e) Loss of CR fibres in SGZ (Grade 1), (f) sprouting fibres in the inner molecular layer (Grade 2). (g) Extensive sprouting in outer molecular layer (Grade 3). (h–k) Neuropeptide Y. (h) Normal pattern with clear definition between the inner and outer molecular layer and absent fibre sprouting (Grade 1), (i) distinction between inner and outer molecular layer fibres visible but increased radial fibre networks (Grade 1), (j) loss of boundary between inner and outer molecular layer and increased radial fibres (Grade 2), (k) as for Grade 2 but increased fibre plexus in SGZ region in addition (Grade 3). (l–n) Calbindin in the dentate gyrus. The patterns of labelling in the granule cells was graded as recently described (Martinian et al., 2012): (l) normal pattern with labelling of majority of granule cell neurones and apical dendrites (Grade 0), (m) loss of CB expression (Grade 1), (n) basal granule cells negative and dispersed granule cells positive (Grade 2). Bar = 45 μm.

The patterns of cellular and synaptic alteration in the dentate gyrus were graded independently on each preparation based on established criteria and previous studies (Blumcke et al., 2009; Magloczky, 2010; Magloczky et al., 2000; Martinian et al., 2012; Nitsch and Ohm, 1995). On CV stained sections, the predominant cytoarchitectural pattern of the granule cells was graded as: normal (Grade 0); mild dispersion (Grade 1); severe dispersion (Grade 2) (Fig. 1a–c). The presence of predominant granule cell loss was also recorded (Blumcke et al., 2009). In CR sections, the pattern of fibre sprouting was graded as: normal with dense bundles of fibres in the immediate inner molecular layer (IML) just above the granule cell layer (GCL) and similar fibres in the sub granular zone (SGZ) (Grade 0) (Nitsch and Ohm, 1995); loss of CR fibres in SGZ (Grade 1); sprouting fibres in the IML (Grade 2); extensive sprouting in IML and outer molecular layer (OML) (Grade 3) (Magloczky et al., 2000) (Fig. 1d–g). In NPY-labelled sections, the sprouting was graded as previously reported (Thom et al., 2009): normal pattern (Grade 0); distinction between IML/OML fibres visible but increased radial fibre networks in GCL and IML (Grade 1); loss of boundary between IML/OML and increased radial fibres in GCL and IML (Grade 2); as for Grade 2 but increased fibre plexus in SGZ region in addition (Grade 3) (Fig. 1h–k). In CB-labelled sections, labelling in the granule cells was graded as recently described (Martinian et al., 2012): normal pattern with labelling of majority of neurones (Grade 0); loss of CB expression (Grade 1); basal granule cells CB-negative and dispersed granule cells positive (Grade 2) (Fig. 1l–n).

### Quantitative methods: pyramidal and interneuronal densities

The following methods were applied to all sections from cases and controls. On CV sections, using *Histometrix* image analysis system (Kinetic Imaging, UK), regions of interest (ROI) were drawn to include CA1 pyramidal cell layer and the area between the blades and boundaries of the dentate gyrus granule cell layer (designated as CA4), with 2.5× objective as previously described (Thom et al., 2005, 2010a). The CA1 ROI was restricted to the stratum pyramidale with care to exclude white matter, CA2 and subiculum at each border. All CV-stained cells with the morphology of pyramidal cell neurones were counted with a 64× objective magnification within these ROI using the optical dissector, a stereological 3D cell-counting method (Thom et al., 2005), with uniform random field sampling and a sampling fraction of between 25 and 100% (mean 50% over all sections; average fields counted 87 in CA1, 102 in CA4), to minimise coefficient of error to 0.1 or less where possible. In CR-labelled sections, similar ROI were drawn. In CA1 a higher density of neurones was noted on the inner border of CA1 with the adjacent strata and an increase in CR fibre networks in CA2 subfield as previously described (Nitsch and Ohm, 1995), which were not included in the ROI. CR-immunopositive cells of all morphologies (Nitsch and Ohm, 1995; Toth et al., 2010) were counted, using ‘2d’ cell profile counting methods, at 40× with a field sampling fraction of 100% in all cases. Cells clearly within the GCL in marginal CA4 fields, dilated processes and varicosities without a nucleus in the section plane, were not counted. In CB-sections, the same method was employed as for CR and all immunopositive cells regardless of size or morphology counted, including hypertrophic neurones in HS (Magloczky et al., 2000). Some pyramidal cells showed weak CB immunopositivity but were not counted. In NPY sections, only immunopositive cells in CA4 ROI were counted due to infrequency of positive cells in the stratum pyramidale of CA1 in the majority of cases. In all cases, in CB, CR and NPY sections, normal labelling of cortical and white matter interneurones was noted as positive internal control, but not quantified. From the left and right hippocampus at the level of, or nearest to, the lateral geniculate nucleus in each case, the size of all CB-positive neurones within CA4 and CA1 was measured using Image Pro Plus software (Media Cybernetics). The mean cell size was recorded (Table 2) and compared between cases.

### Statistical analysis

Statistical analysis was carried out using SPSS version 16. The Kruskal–Wallis test was used for comparison of neuronal densities between post mortem groups and Pearson's correlation between neuronal subtypes and groups; *p* < 0.05 was considered as significant.

## Results

### Neuronal loss over all epilepsy cases

The analysis was carried out on a range of 3–6 (mean 4.1) of the seven anatomical levels per case. Different levels were available between cases but included at least one block from anterior, middle and posterior hippocampus in all cases except one (case EP254 a patient with a previous temporal lobectomy where anterior sections were not available). Analysis of all sections from epilepsy cases, sides and levels showed a significant correlation between pyramidal cell neuronal density as quantified with CV and interneuronal numbers for CR (*p* < 0.01), CB (*p* < 0.0001) and NPY (*p* < 0.005) in CA4. This suggests that loss of principal cells occurs in parallel with interneuronal loss in CA4. In CA1, subfield a significant correlation was noted only between CV and CB neuronal densities (*p* < 0.0001) (Table 2).

**Figure 2:**
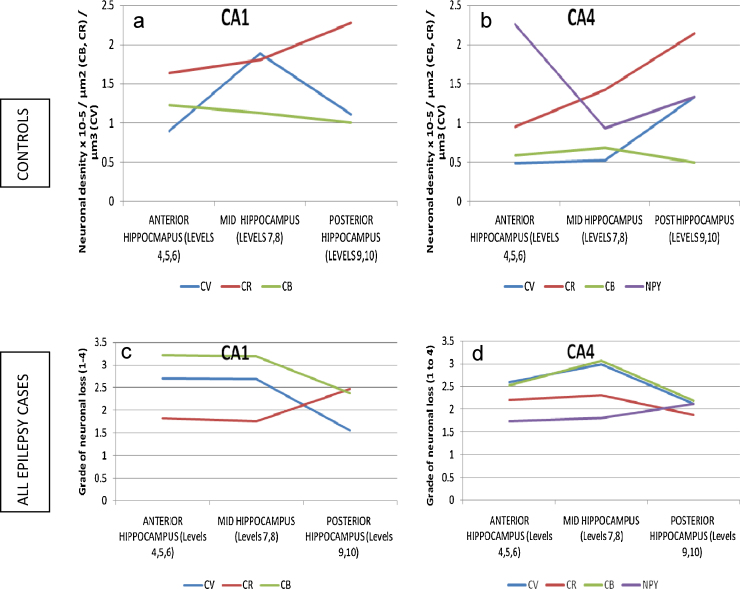
Anterior posterior variability of neuronal densities in controls and neuronal loss in epilepsy cases. Trend lines for variation of control data values (mean neuronal densities), top row for CA1 (a) and CA4 (b) along the hippocampal axis for pyramidal cells (CV), calretinin (CR), calbindin (CR) and NPY-positive interneurones. Bottom row shows mean severity of neuronal loss in all epilepsy cases expressed as four grades (1–4) for the severity of neuronal loss (grade 4 being 75% or greater neuronal loss; see main text for details) in CA1 (c) and CA4 (d) along the hippocampal axis. In the controls for CV increased neuronal density is noted for the mid levels compared to anterior, but less so for CA4. Calretinin positive neurones show a progressive increase in neuronal density in an anterior–posterior direction in both CA1 and CA4 whereas calbindin positive neurones remain relatively constant through levels. NPY neuronal densities showed greater variability throughout levels. In epilepsy cases, the relative loss of calretinin and NPY interneurones was less than CV pyramidal cells; CB showed equal or greater neuronal loss in CA1 and CA4. Apart from CR values in CA1 (and to a minor extent NPY), there was a trend for greater neuronal loss for pyramidal cells and interneurones in the anterior and mid hippocampal than for posterior levels. The severity of neuronal loss for pyramidal cells and calbindin in CA4 showed a very close correlation.

In control cases, variability of neuronal densities for CA1 and CA4 were noted along the longitudinal axis of the hippocampus (Table 1 – supplemental) with increased pyramidal (CV) neuronal densities in mid hippocampal levels compared to anterior, but less so for CA4 as previously noted (Dam, 1980) (Fig. 2). CR-positive neurones showed a progressive increase in neuronal density in an anterior–posterior direction in both CA1 and CA4. CB-positive neurones remain relatively constant through levels. NPY neuronal densities showed greater variation through all levels. Due to this normal anatomical variation, counts in epilepsy cases in CA1 and CA4, as quantified on CV, CB, CR and NPY at each level were compared to mean control values for that level and then expressed as four grades: 0–25% neuronal loss (Grade 1), 25–50% neuronal loss (Grade 2), 50–75% neuronal loss (Grade 3) and 75% or greater neuronal loss (Grade 4). In nine of the ten epilepsy cases (all except EP254) neuronal loss of 75% or greater with CV was seen in at least one level in at least one subfield.

In epilepsy cases overall, the relative loss of CR and NPY positive interneurones was less than principal pyramidal neuronal loss in CA1 and CA4, whereas equal or greater relative loss of CB-positive interneurones was noted (Fig. 2). Apart from CR values in CA1, there was a trend for greater overall relative neuronal loss of both pyramidal cells and interneurones in the anterior and mid hippocampal than in posterior levels (Fig. 2). The severity of pyramidal and CB cell loss in CA4 showed a very close correlation with each other throughout the hippocampal axis. Although enlarged CB-positive cells of up to 685 μm^2^ area were observed in epilepsy cases, there was no correlation between mean neuronal size and density in epilepsy cases (Table 2).

### Distribution and variability of longitudinal sclerosis between cases

**Figure 3:**
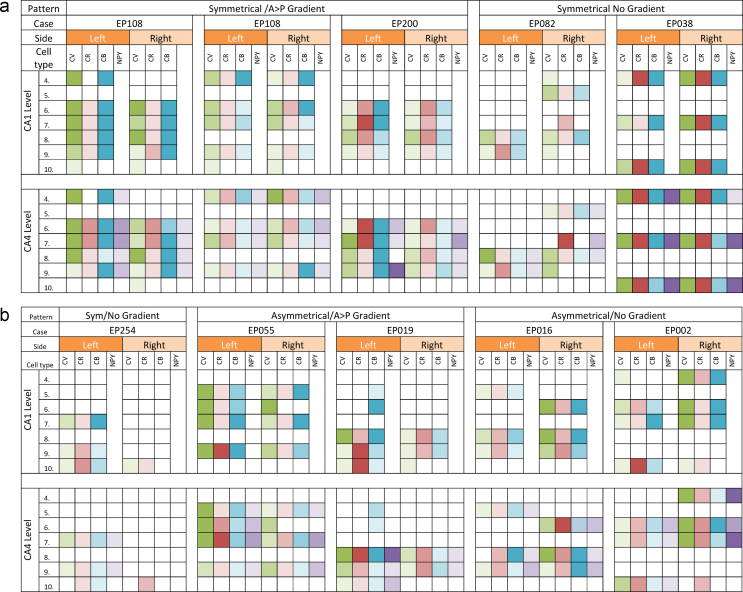
Distribution of the cell loss in left and right hippocampus from CA1 and CA4 in each case at different levels (4–10) along the longitudinal axis. Each neuronal cell type in a different column is depicted with a different colour and shown in four shades corresponding to the grade of neuronal loss. The darkest shade represents >75% loss of cells relative to control values at that hippocampal level, the next darkest shade 50–75% loss of cells, the next darkest shade 25–50% loss of cells and 0–25% neuronal loss is depicted in the lightest shade. (White cell corresponds to no section available for that level.) Cresyl Violet (CV) is shown in green, calretinin (CR) in red, calbindin (CB) in blue and NPY in purple. Level 4 is the most anterior most block and 10 is the hippocampal tail as detailed in text. Based on patterns the cases were grouped into four patterns (see Table 2): (1) symmetrical hippocampal sclerosis (HS) with anterior–posterior (AP) gradient (3 cases), (2) symmetrical HS without AP gradient (3 cases), (3) asymmetrical HS with AP gradient (2 cases) and (4) asymmetrical cases without AP gradient (2 cases). (For interpretation of the references to color in this figure legend, the reader is referred to the web version of the article.)

**Figure 4:**
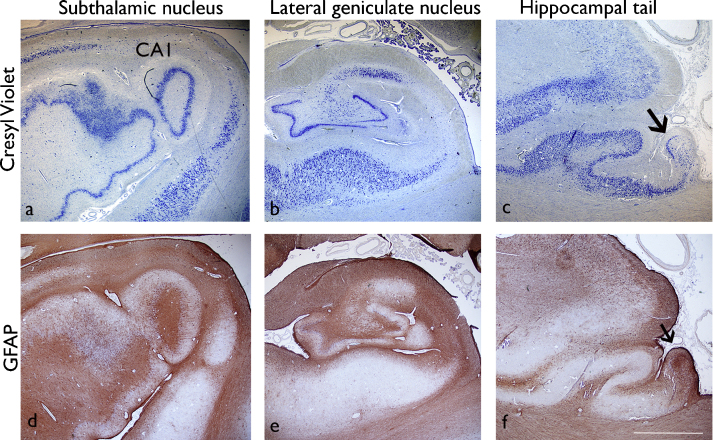
Symmetrical hippocampal sclerosis with an anterior–posterior gradient (Case 1/EP108). Left hippocampus with Cresyl Violet (a–c) GFAP (d–f) shown across three hippocampal levels from the subthalamic nucleus to the tail. The arrows in (c and f) indicate the position of the dentate gyrus and CA4 in the tail. In this case there was a gradient for neuronal loss with more severe neuronal loss (and gliosis) in CA1 and CA4 anteriorly compared to the hippocampal tail. Bar = 1000 μm.

**Figure 5:**
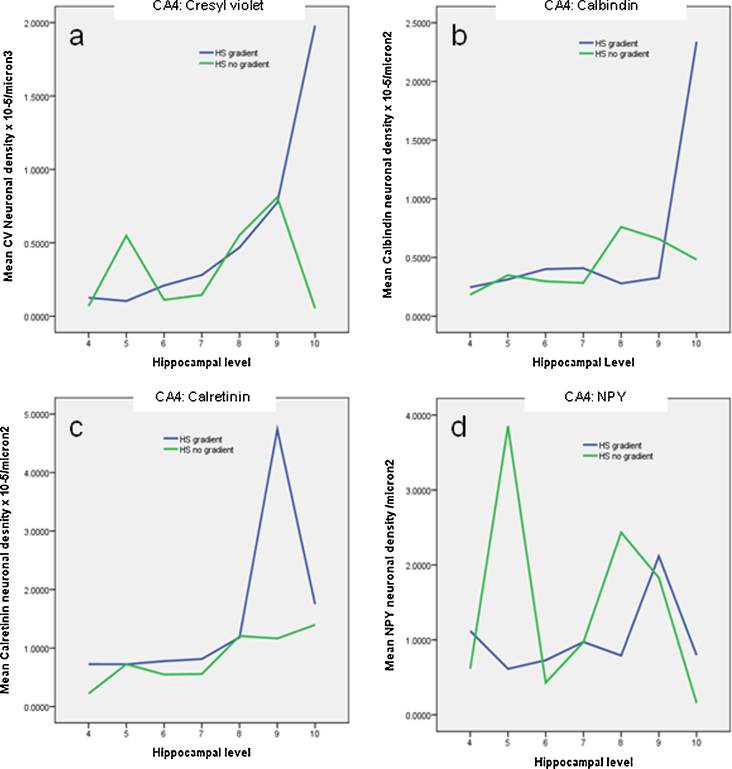
Line graphs shown for mean CA4 neuronal densities with Cresyl Violet (a), calbindin (b), calretinin (c) and Neuropeptide Y (d) plotted against hippocampal levels 4–10 in an anterior, posterior gradient (see text for details of method). Five cases with an anterior–posterior gradient are grouped together (Groups 1 and 3, cases 1, 2, 3, 7 and 8; see text) and five cases without a gradient for neuronal loss (Groups 2 and 4, cases 4, 5, 6, 9 and 10). In the cases with asymmetrical HS only values from the more sclerotic hippocampus were included. The graphs confirmed that in cases with gradients, higher mean neuronal densities were seen in the posterior levels (9, 10) compared to anterior (4–6). The NPY appears less consistent through levels.

The four grades of neuronal loss defined above were plotted to view the variability and distribution of cell loss along the longitudinal hippocampal axis and between left and right sides in individual cases (Fig. 3). Cases were judged as having an asymmetrical pattern of neuronal loss between left and right hippocampus if there was a difference in neuronal loss at a level of two grades or more (for example cell loss of 0–25% on the right and 50–75% on the left), in the majority of levels examined in the case. Similarly, cases were regarded as showing a gradient of cell loss along the longitudinal axis if a difference in neuronal loss of two grades or more was noted between any two levels on one side. The direction of the gradient of cell loss was also noted, for example anterior versus posterior predominance. This analysis was carried out for all neurones and interneurones and tabulated (Table 2 supplementary table) and four groups emerged based on predominant patterns: (1) symmetrical HS with anterior–posterior (AP) gradient (3 cases) (Fig. 4) (2) symmetrical HS without AP gradient (3 cases), (3) asymmetrical HS with AP gradient (2 cases) and (4) asymmetrical cases without AP gradient (2 cases). In the majority of cases in Groups 1 and 3 (4/5 cases), an anterior > posterior gradient was predominant although not consistent for all neuronal types (Table 2 supplementary table). In support of this grouping, statistical analysis showed significant differences in CV neuronal densities between AP levels but not left and right hemispheres in Group 1 (symmetrical/gradient) for both CA1 (*p* = 0.01) and CA4 (*p* = 0.018); similarly between left and right hemispheres but not AP levels in Group 4 (asymmetrical/no gradient) for CA1 (*p* = 0.006) and CA4 (*p* = 0.055). The differences for interneuronal densities was less consistent, with significant differences noted for CR-positive neurones in Group 1 between AP levels in CA4 (*p* = 0.01) and CB-positive neurones in CA1 in Group 4 between hemispheres (*p* = 0.016). There were no significant differences for NPY-positive interneurones in these groupings, which showed greater variability between levels (Fig. 5).

Studies of HS in surgical samples define subtypes or patterns of sclerosis as either classical or atypical based on the distribution of pyramidal cell loss (Blumcke et al., 2007; Thom et al., 2009). Based on our quantitative data in CV stained sections, the HS subtype in PM cases was designated as classical HS (CHS) (50% or greater neuronal loss from both CA4 and CA1), CA1 predominant HS (CA1p) (50% or greater neuronal loss from CA1 alone) or end folium sclerosis (EFS) (50% or greater neuronal loss from CA4 alone) for each section. The GFAP-stained sections were used to confirm pattern of regional gliosis and sclerosis (Fig. 4d–f). The variations of patterns of sclerosis along the longitudinal axis and between hemispheres were tabulated (Table 3). Based on these criteria, in only 6 of the 20 (left and right) hippocampi, did the pattern of sclerosis (or its absence) remain constant in all levels examined. In 8 of the 10 cases, HS extended in to the tail (levels 9 and 10), with CHS in one case (EP038) and atypical patterns in the remainder (Table 3).

### Dentate gyrus reorganisation

**Figure 6:**
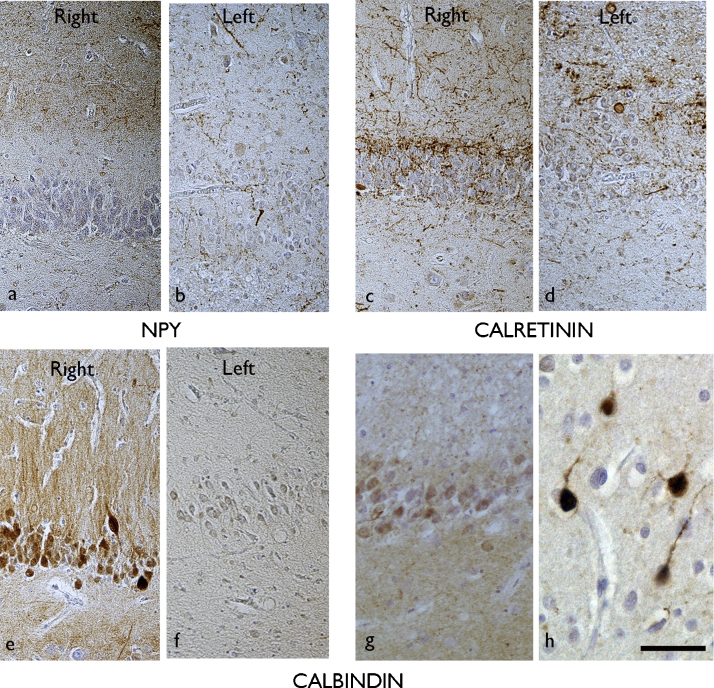
Hippocampal sclerosis with interneuronal changes extending into the hippocampal tail. Hippocampal sections are shown at the level of the hippocampal tail for (EP200) with NPY (a) = right, (b) = left; calretinin (c) = right, (d) = left; calbindin (e) = right, (f) = left. Normal dentate gyrus interneuronal patterns were observed in the right side with sprouting (NPY, CR) or loss of expression (CB) noted on the left side, supporting asymmetrical alteration. EP038 demonstrated bilateral alterations extending into the tail, with loss of calbindin in the granule cells shown in (g) in the hippocampal tail section on the left; the adjacent cortex showed normal pattern of cortical interneurons on the section confirming the technical efficacy of the staining. Bar = 55 μm.

Over all sections, there was a significant correlation between the grades of axonal reorganisation in the dentate gyrus granule cell and molecular layer for CR and NPY (*p* < 0.001) (Fig. 6). There was also an inverse correlation between the grade of sprouting observed with CR labelling and CA4 neuronal densities with CV (*p* < 0.0001) and CR (*p* < 0.01) labelling, and similarly for NPY fibre sprouting and CA4 neuronal densities with CV (*p* < 0.001) and NPY (*p* < 0.05), supporting a relationship between hilar neuronal loss and sprouting. There was a significant negative correlation between CB expression patterns in the dentate gyrus granule cells and CA4 neuronal densities as measured with CV (*p* < 0.05) and positive correlation with CR and NPY-labelled fibre sprouting (*p* < 0.001).

Within cases, the grades of GCD and reorganisation with CB, CR and NPY were plotted to view the variability and distribution along the longitudinal hippocampal axis and between left and right sides (Supplemental Fig. 1). Asymmetry for dentate gyrus reorganisation and GCD was considered as present when a difference of 2 or more grades was observed between left and right side at a single level, for more than half the levels (Fig. 6a–f); A gradient was considered as present if similar differences in grades between levels were present in one hemisphere. This was tabulated and overall the distribution of pathology aligned with the four groups pre-determined by the pattern of neuronal loss (Supplementary Table 2). There was no significant difference between the four groups regarding the severity of fibre reorganisation apart from CB in Group 2 (symmetrical/no gradient, *p* < 0.02), where no sections showed Grade 2 pattern for CB (Fig. 1n), compared to 37.5%, 29% and 31% of sections in Groups 1, 3 and 4 respectively. In individual cases we confirmed pathological re-organisation extending into the posterior hippocampal tail levels, with GCD observed in cases (EP038, EP055), and NPY, CB and CR reorganisation in (EP108, EP019 and EP016/EP038 respectively).

### Clinical–pathology correlation

In the epilepsy patients the mean age at death was 74 years (Table 1). The mean age of onset was 8.5 years and the duration of epilepsy 60.5 years, with some patients having prolonged seizure-free intervals prior to death. In only one patient was the syndrome of mesial temporal lobe epilepsy diagnosed and in this patient (EP054), bilateral reduction in neurones and interneuronal sprouting were observed, with changes predominating in anterior and mid hippocampal blocks. The series is too small for extensive clinico-pathological correlation but of note, in four of five patients, clinical information was available and that showed a gradient for HS indicating more focal hippocampal pathology, seizure onset was at 18 months of age or earlier. The two patients with significant and bilateral neuronal loss with no gradient, supporting more diffuse and widespread damage (Group 2; EP082, EP038), had later seizure onsets of 13 years and 15 years. We included two patients (EP016, EP254) who had undergone epilepsy surgery decades prior to death at other institutions, with partial removal of the right temporal lobe and hippocampus confirmed at post mortem; both continued to have seizures post-operatively. We were unable to trace the surgical specimens in either case. Although fewer levels were available, we confirmed residual typical unilateral CHS extending into the tail on the operated side and interneuronal sprouting and GCD compatible with epilepsy-mediated sclerosis rather than post-surgical scarring of the remnant in EP016. In EP254, no significant neuronal loss or interneuronal sprouts were identified in the hippocampal remnant or contralateral side, but frontal lobe focal cortical dysplasia was identified. In this series of elderly patients with epilepsy, a variable degree of pathological tau accumulation was noted with low Braak stages (0–III) in five cases and mid Braak stages (III–IV) in five; no high Braak stages were seen. In all the mid Braak stage cases, typical interneuronal sprouting was present in all, with striking asymmetry of neuronal loss in two patients, suggesting epilepsy-mediated hippocampal atrophy.

## Discussion

We have utilised a post mortem epilepsy series to explore the variability in HS along the length of the hippocampal body and tail, including alterations to interneuronal populations, in patients with epilepsy. This small series of cases demonstrated a marked longitudinal variability in patterns of damage. Whilst case numbers are small, four patterns appear to emerge and we have confirmed for the first time that in some cases pathology extends into the hippocampal tail. Modern neuropathological studies of HS in epilepsy have largely focused on surgical tissue through examination of this process in one coronal plane, and have considered this one section as representative of the pathology. Our findings suggest this may be an over simplification. These less explored aspects of both the longitudinal extent and variability of HS may provide explanations for poor outcome following surgery, as well as discrepancies between surgical series with clinico-pathological correlations (Thom et al., 2010a).

Margerison and Corsellis’ seminal post mortem study of 55 patients with temporal lobe epilepsy identified bilateral patterns of sclerosis in 31% of patients, but did not consider patterns of atrophy along the longitudinal axis (Margerison and Corsellis, 1966). This axis was studied in a series of 20 brains from patients with epilepsy (Mouritzen Dam, 1982), including patients with long epilepsy histories of 30 years or more. A mean of 3.5 sections through the middle part of the hippocampus was quantified for subfield neuronal loss, but the precise anatomic levels included in this study were not detailed, the tail of the hippocampus was not included and only pyramidal neurones and granule cells were counted. This study identified greater loss of neurones in the anterior hippocampus, showing a greater relative reduction than the normal increasing anterior to posterior gradient of densities seen in controls. In a surgical series of 12 resected hippocampi, comparison between the anterior and posterior resection margins showed greater anterior neuronal loss in a subgroup, but more diffuse neuronal loss throughout the hippocampus in others, this latter pattern being associated with multifocal EEG spiking (Babb et al., 1984b). Sloviter et al. carried out detailed analysis of hippocampal surgical resections in temporal lobe epilepsy utilising serial sections to compare patterns of sclerosis with hippocampal convolutional malformations. No detail of gradients of neuronal loss was provided, and this was not the primary purpose (Sloviter et al., 2004). Experimental data also provide information on the regional distribution of neuronal loss. In the lithium pilocarpine-induced status epilepticus model, greater neuronal loss was observed in the ventral hippocampus in CA1 following acute seizures in immature animals (Ekstrand et al., 2011). Greater susceptibility of the ventral hippocampus, particularly in early lesions, has also been demonstrated with exposure to other seizure-inducing agents (Apland et al., 2010), in support of regional differences.

We aimed to align coronal levels, to compare to similar levels in controls and extend the evaluation to the most posterior extent of the hippocampus. We undertook quantitative immunohistochemistry for interneuronal markers which have been extensively studied in surgical HS cases as additional evidence for epilepsy-induced hippocampal damage. We proposed four patterns based on asymmetry (or not) of sclerosis between hemispheres [as previously well-recognised (Margerison and Corsellis, 1966; Meencke et al., 1996; Thom et al., 2009)] and the presence of either more diffuse sclerosis versus a gradient for pathological changes along the longitudinal axis. In keeping with previous observations by Dam and Babb (Babb et al., 1984b; Dam, 1980) looking at mean values over all our epilepsy cases, greater anterior hippocampal damage was seen. Within individuals, we did identify cases where pathological changes were more evenly distributed and diffuse, including alterations to interneuronal subsets and with equal involvement of the hippocampal tail.

In quantitative and volumetric MRI studies of patients with temporal lobe epilepsy that have addressed the longitudinal distribution of changes, both focal and diffuse patterns of volume loss have been identified (Bronen et al., 1995; Kuzniecky et al., 1996; Woermann et al., 1998). Quigg et al. (1997), in a volumetric MRI study of 43 TLE patients with HS compared to controls, proposed that the process was overall diffuse, not favouring the head or tail. In none of these series were MRI changes correlated with extent of neuropathology findings in resected specimens. Our current post mortem series is compromised by small case numbers, and mainly non-TLE syndromes. Nevertheless, we formed the impression that focal hippocampal pathology was associated with early childhood onset of seizures (with an initial precipitating injury (IPI) of febrile seizures in one case at 18 months), whereas bilateral, diffuse damage was observed with adolescent onset of seizures (with later IPI, in one case at 13 years). An MRI study of 47 cases of TLE and HS found hippocampal tail atrophy was associated with seizure onset before age 10 years (Kuzniecky et al., 1996). In a further study of 16 TLE patients, younger age of seizure onset (median 2.5 years) was noted in patients with bilateral HS, compared to unilateral diffuse (median 7.5 years) and unilateral and focal HS (median 14 years), but with wide age ranges within these groups (Woermann et al., 1998). These inconsistent findings indicate that larger series need to be studied with clinical, radiological and pathological correlation. Factors other than age of onset of seizures likely play a role and need to be considered in order to understand an individual's susceptibility for diffuse versus focal HS.

In a previous quantitative study of interneurones in surgical HS specimens, a reduction of ∼50% of CR-positive neurones was shown, particularly in the CA4 or hilar region (Magloczky et al., 2000). A reduction of 76.1% of CA1 CR neurones and 32.1% of CA4 neurones was shown, correlating with the severity of principal neuronal loss (Toth et al., 2010). Early studies supported preferential survival of interneurones in HS, including CB-positive cells (Sloviter et al., 1991). Reduction of CB-positive interneurones has since been confirmed in CA1 (Magloczky, 2010; Wittner et al., 2002), with increased size of the cell body (Magloczky et al., 2000). Studies in hippocampal sclerosis also support a selective vulnerability of hilar NPY-positive neurones (Mathern et al., 1995). In our post mortem series, we confirm loss of interneurones of all types in parallel with principal cell loss, particularly in the CA4 region, with less consistent patterns for CA1. We also demonstrated a greater relative vulnerability of CB-positive interneurones, in contrast to previous studies (Sloviter et al., 1991). Furthermore, we confirmed that loss of interneurones may be present along the length of the hippocampus.

It has been argued that axonal sprouting is a more relevant process in the epileptogenic hippocampus (Magloczky, 2010). Sprouting particularly affects NPY- and CR-fibre networks, with comparable and well-defined stages of re-organisation (Magloczky, 2010; Magloczky et al., 2000; Mathern et al., 1995; Thom et al., 2009). We confirmed that patterns of fibre sprouting correlated with hilar neuronal loss overall cases. Within our four groups, data on asymmetry and gradient of sprouting were less consistent, but the numbers in each group were small. However, pathological alterations were observed along the hippocampal length extending into the hippocampal tail in some cases. We have also recently reported on distinct patterns of restricted absence of CB expression from basal granule cells in HS (Martinian et al., 2012) and that this may relate to early onset of seizures and impaired normal maturation (Abraham et al., 2009). In the present series, we showed similar alteration in CB expression in granule cells, even involving sections from the hippocampal tail. We also confirmed the lack of this pattern in Group 2 (symmetrical HS, no gradient), which may relate to their later onset of seizures.

One rationale for the study of the longitudinal extent of HS and confirmation of focal versus diffuse damage is that this might influence post-operative outcome after temporal lobectomy. In an MRI series of 57 patients, the extent of hippocampal volume reduction did not influence outcome (Bronen et al., 1995), a finding replicated in a further study of 43 patients (Quigg et al., 1997). However, patients with more extensive neuronal loss in the resected hippocampal specimen had less benefit from surgery compared to those with greater anterior sclerosis as confirmed by neuropathology (Babb et al., 1984a). Surgical resections that extend more posteriorly are more beneficial, with a significantly greater proportion of patients becoming seizure-free (Wyler et al., 1995) although in recent large series this relationship is less clear (Helmstaedter et al., 2011b; Schramm, 2008; Schramm et al., 2011). The risk of extended hippocampal resection is greater impairment of memory post-operatively (Helmstaedter et al., 2011b), particularly where the pathological abnormalities are less severe (Helmstaedter et al., 2011a). The extent of post-operative shrinkage of the remnant of hippocampus, which can be independent of presence of HS, also influences post-operative memory decline (Baxendale et al., 2000; Mueller et al., 2009). All these factors have to be considered in the pre-surgical approach and planning in any individual. In the present series, we included two patients who had undergone previous epilepsy surgery: in one, residual specific patterns of CHS were present in the remnant on the operated side. Furthermore, even in our cases with a gradient of HS (indicating more focally severe, often anterior damage), subtle degrees of neuronal loss, atypical HS and synaptic changes were still noted in the hippocampal tail. This would correspond to the surgical remnant following a standard anterior temporal lobe resection and arguably could act as a nidus for continued seizures. Our previous studies of surgical HS along the longitudinal axis have shown that HS usually extends throughout the specimen to the surgical resection margins (Thom et al., 2002). By inference, this would imply residual HS in the adjacent remnants. Experimental data has shown that along the longitudinal axis there is enhanced neurogenesis observed adjacent to the sclerotic segment which may be pro-epileptogenic (Haussler et al., 2011).

The ‘lamellar’ hypothesis, proposing that segments of the hippocampus were functionally independent, was questioned by the demonstration of extensive intrinsic longitudinal connections, functionally relevant to information processing (Amaral and Witter, 1989). Nevertheless, modern anatomical information from animals demonstrates that CA1 outputs along the longitudinal hippocampal axis provide precise regional cortical organisational patterns supporting differential functional properties (Cenquizca and Swanson, 2007; de Hoz et al., 2003). Evidence of differences in timing of neuronal maturation along the longitudinal axis of the hippocampus (Piatti et al., 2011) as well evidence for different age-related vulnerabilities (Ta et al., 2011), may all be relevant to patterns of focal versus diffuse neuronal loss observed in HS in epilepsy and resulting clinical impairments. Furthermore, a recent experimental study has confirmed the importance of longitudinal hippocampal projections in the spread of seizures which may be relevant to human hippocampal seizures (Pallud et al., 2011).

Our post mortem epilepsy series has limitations. A variety of syndromes are represented and the findings are not directly comparable to surgical series. In our previous studies we demonstrated that mossy fibre sprouting may be a bilateral epiphenomenon and occur in epilepsy syndromes other than TLE (Thom et al., 2009). We now demonstrate that similar alterations to CR and CB expression are seen to those previously only described in TLE patients and that these alterations are not syndrome-exclusive. Longer fixation times for post mortem tissues are a further limitation, compared to short fixation times for surgical tissues which precluded study of some antigens, such as dynorphin. We therefore included control cases with comparable fixation times and our CR cell densities are a similar range to those in a previous post mortem study (Toth et al., 2010). The controls that were available were younger and age-related reduction in hippocampal neuronal densities of up to 20% can occur from age 68 onwards (Mouritzen Dam, 1982; Simic et al., 1997). We set a cut-off point of greater than 50% neuronal loss for the diagnosis of HS. In 9 of the 10 cases (all except EP254), we observed >75% pyramidal cell neuronal loss in at least one subfield at one level. In addition, in our older epilepsy cohort, the possibility of co-existing ischaemic hippocampal damage or atrophy as a result of aging alone, could contribute to the severity of sclerosis (Nelson et al., 2011; Pao et al., 2011). However, in favour of a predominant epilepsy-mediated pattern of atrophy, the interneuronal sprouting observed is similar to that reported in surgical samples from young epilepsy patients and there was an absence of subicular atrophy, often present in HS associated with aging. Neurofibrillary tangles were present in the hippocampi, although the Braak stage was not high in any case. We have previously demonstrated that hippocampal sclerosis in epilepsy likely precedes tau accumulation (Thom et al., 2011). Nevertheless, we cannot exclude that accelerated aging, including Alzheimer's disease pathology, has contributed to some of the hippocampal neuronal loss observed in our cohort.

In summary, we have confirmed that the pathological changes of HS are variable along the longitudinal axis and restriction of the examination to one hippocampal level may not accurately represent the whole process. In addition, the pathology can extend into the hippocampal tail in some patients with epilepsy. We have demonstrated that alterations to interneuronal populations previously described unilaterally in surgical specimens can occur bilaterally and extensively and are not syndrome-exclusive. More widespread asymmetrical and diffuse hippocampal damage may be relevant to surgical failure in TLE patients. Further studies are required to explore the clinical factors that influence focal versus diffuse HS.

## Figures and Tables

**Table 1:** Clinical data of cases and controls.

#	Case number	Age	Sex	IPI	Syndrome	Onset epilepsy [months (m), years (yr)]	Duration of epilepsy (recent seizure pattern)	Seizure types	Associated neuropathology (other than HS) Braak stage^a^	Cause of death
1	EP108	53	F	Unknown	Unknown	12 m	52 years. (Drug resistant lifelong epilepsy)	CPS, SGTCS	None Braak stage = 0	SUDEP
2	EP054	71	F	Birth injury and FS at 18 months with pneumonia	mTLE	10 m	65 years. (Seizure free for 5 years before death)	SE, SPS, CPS, SGTCS	Cerebellar atrophy Braak stage = 3	PE
3	EP200	79	M	Unknown	Unknown	4 m	Unknown	Unknown	Lacunae infarcts Braak stage = 4	Acute haematemesis
4	EP082	82	F	None	Unknown	15 yr	Unknown	SPS, CPS, SGTCS	Cerebellar atrophy Small infarct Braak stage = 0	
5	EP038	74	M	Uncertain-possible IPI age 13	Symptomatic focal	13 yr	61 years. (Seizures up to 4 months before death)	CPS, SGTCS	Hemi-cortical atrophy Cerebellar atrophy Old contusions Braak = 4	GI haemorrhage
6	EP254	59	F	No	Symptomatic focal	3 yr	56 years (CPS continued/No SGTCS 9 years before death)	SPS, CPS, SGTCS	Previous right temporal lobectomy FCDIIB (Frontal) Braak stage = 2	Lung tumour
7	EP055	97	F	Seizures following childhood smallpox	Syndromic classification not possible	?	Uncertain (epilepsy in remission prior to death)	CPS, SGTCS	Old lacunae infarct. Cerebellar atrophy. Braak stage = 4	Aspiration pneumonia
8	EP019	74	M	Encephalitis 18 month following vaccination	Unknown	18 m	73 years (Minor seizures continued to death. No remission)	SPS, CPS, ?SGTCS	Lacunae infarct thalamus Old hemicortical atrophy Cerebellar atrophy Braak stage = 2	PE
9	EP016	75	F	Unknown	Unknown	Infancy	Unknown	GTCS	Previous partial right temporal lobectomy (age 41) Braak stage = 3	Unascertained
10	EP002	76	F	Febrile convulsions	Unknown	Neonatal	Unknown	CPS SGTCS MJ	Ganglioglioma Lacunar infarct in basal ganglia Braak stage = 2	Sudden Cardiac death?
1	Controls	57	F	Not applicable						Pancreatitis
2		38	F							MI
3		58	M							MI

a Braak staging as detailed in Thom et al. (2011). IPI, initial precipitating injury; FS, febrile seizure; mTLE, mesial temporal lobe epilepsy; CPS, complex partial seizures; SGTCS, secondary generalised tonic clonic seizures; SPS, simple partial seizures; MJ, myoclonic jerks. SUDEP, sudden and unexpected death in epilepsy; PE, pulmonary embolus, MI, myocardial infarction, CVD, cerebrovascular disease.

**Table 2:** Mean values for neuronal counts with each stain or antibody over all hippocampal levels for CA1 and CA4 in the left and right hippocampi and mean neuronal cell size with calbindin at the lateral geniculate nucleus.

Pattern of hippocampal sclerosis		Left:right Cresyl Violet CV (pyramidal cells) ×10^−5^/μm^3^		Left:right calretinin CR (interneurones) ×10^−5^/μm^2^		Left:right calbindin CB (interneurones) ×10^−5^/μm^2^		Left:right calbindin cell size μm^2^		Left:right Neuropeptide Y NPY ×10^−5^/μm^2^
Groups	Case	CA1	CA4	CA1	CA4	CA1	CA4	CA1	CA4	CA4
Symmetrical gradient	EP108	0.39:1.7	0.24:0.94	3.65:4.5	1.44:2.15	0.078:0.1	0.15:0.89	32.6:16.9	:96.4	0.71:1.32
	EP054	0.66:0.89	0.71:0.40	4.6:4.0	2.9:2.6	0.82:0.6	0.72:0.32	19.38:16.6	86.12:121.8	2.11:1.12
	EP200	1.16:1.15	0.24:0.70	1.84:1.32	2.73:1.07	0.4:1.0	0.05:0.64	:31.6	:57.1	0.55:1.46
Symmetrical no AP gradient	EP082	1.24:1.02	0.57:0.59	1.3:1.0	1.4:0.8	0.81:0.56	0.69:0.91	102.8:35.9	139:81.9	2.2:3.2
	EP038	0.68:0.09	0.06:0.05	0.68:0.15	0.25:0.08	0.24:0.07	0.14:0.14	:68.9	25.0:14.16	0.08:0.69
	EP254	2.37:2.21	0.60:	1.9:3.3	1.9:1.9	0.56:	0.94:	28.3:	69.7:	2.9:
Asym-metrical AP gradient	EP055	0.14:0.49	0.18:0.38	1.25:2.22	0.86:1.13	0.14:0.1	0.34:0.45	154.5:82.2	106.7:70.6	0.91:0.77
	EP019	1.44:1.80	0.49:1.07	0.65:1.19	1.2:0.64	0.54:0.72	0.51:0.62	21.3:45.3	151.9:81.2	1.46:1.28
Asym-metrical no AP gradient	EP016	1.66:0.25	0.67:0.62	1.45:1.21	1.01:0.63	0.67:0.25	0.37:0.25	38.5:61.9	45.7:60.9	0.85:0.63
	EP002	1.03:0.32	0.87:0.07	1.26:2.03	0.91:0.91	0.39:0.03	0.81:0.23	56.4:	93.8:162	1.03:0.15

**Table 3:** Variations in the patterns of hippocampal sclerosis (HS) along the longitudinal axis as based on quantitative evaluation compared to control data for that level. Pattern of sclerosis was classified as follows: 50% loss or more of neurones on Cresyl Violet section at one level from both CA4 and CA1 (classical hippocampal sclerosis [CHS]); 50% loss or more from CA1 alone (CA1 predominant sclerosis [CA1p]); 50% loss or more from CA4 alone (end folium sclerosis [EFS]). Levels 4–10 as detailed in text.

Number	Case PM number	Side	Hippocampal level Body → tail						
			4	5		7	8	9	10
1	EP108	L	CHS	–	CHS	CHS	CHS	CA1P	No HS
		R	–	–	CHS	CA1p	CHS	No HS	No HS
2	EP054	L	CA1P	–	CA1P	CHS	–	No HS	No HS
		R	EFS	–	CA1P	CHS	–	No HS	No HS
3	EP200	L	–	–	No HS	EFS	CHS	EFS	–
		R	–	–	No HS	No HS	CA1p	No HS	–
4	EP082	L	No HS	No HS	–	–	CHS	No HS	–
		R	No HS	CA1p	–	–	EFS	EFS	–
5	EP038	L	EFS	–	–	EFS	–	–	CHS
		R	CHS	–	–	CHS	–	–	CHS
6	EP254	L	–	–	–	No HS	–	No HS	No HS
		R	–	–	–	–	–	–	No HS
7	EP055	L	–	CHS	CHS	CHS	–	CA1P	–
		R	–	No HS	CHS	No HS	–	CHS	–
8	EP019	L	–	–	–	–	CHS	EFS	No HS
		R	–	–	–	–	No HS	No HS	No HS
9	EP016	L	–	No HS	–	–	No HS	No HS	–
		R	–	–	CHS	–	CHS	CA1P	–
10	EP002	L	No HS	–	No HS	No HS	–	–	EFS
		R	CHS	–	CHS	CHS	–	–	–

## Appendix A Supplementary data

Figure 1 (Supplemental file) Dentate gyrus reorganisation in HS cases observed with calretinin, calbindin and Neuropeptide Y (NPY) and the pattern of granule cell dispersion (GCD) along the hippocampal axis. Each is depicted in a different colour which is shown in four shades and corresponds to the grade of alterations. Calretinin (green): Grade 0 = normal pattern (palest shade), Grade 1 = reduction of infragranular fibres/increase in supragranular fibres and reduction in infragranular fibres (mid shade), Grade 2 = supragranular sprouts in the inner molecular layer (IML) (darker shade), Grade 3 = extensive fibres in IML and outer molecular layer (OML) (darkest shade). Calbindin (Orange): Grade 0 = normal pattern (palest shade), Grade 1 = calbindin mainly lost in granule cells (mid shade), Grade 2 = Basal granule cells calbindin negative, distal cells calbindin positive (darkest shade). NPY (Purple): Grade 0 = normal (palest shade), Grade 1 = some increase in fibres in IML with maintenance of difference between IML/OML (mid shade), Grade 2 = marked sprouts in IML and OML (darker shade), Grade 3 = sprouts in OML and infragranular region (darkest shade). GCD (blue) is graded as predominant cell loss (lightest shade), normal = Grade 0 (mid shade), Grade 1 = mild dispersion (darker shade) and Grade 2 = severe dispersion (darkest shade). The changes are shown in the left hippocampus (top row) and right hippocampus (bottom row) along the anterior to posterior longitudinal axis of the hippocampus from levels 4 to 10 (see text for details of levels).

Table 1 (Supplementary table) Mean control values for neuronal densities at different levels for the left and right hippocampus from three controls (six hippocampal specimens) (*NPY stained sections not available for these levels but from next anterior level).

Table 2 (Supplementary table) Four groups of hippocampal sclerosis (HS) based on the predominant pattern of left: right asymmetry and the gradient of neuronal and interneuronal loss along the longitudinal axis. Asymmetry was defined as a difference of 2 grades** between left and right sides for more than 50% of levels examined. An A-P gradient was defined as a difference of 2 grades** or more between any levels from one hemisphere. [A = cell loss predominant in anterior levels (levels 4–6), P = cell loss predominant in posterior levels (9, 10) and M = cell loss predominant in mid levels (7, 8)]. *Limited levels from this case as post. temporal lobectomy. **The four grades were 0–25%, 25–50%, 50–75% or 75%+ of neuronal loss relative to control values for that level. Asymmetry for dentate gyrus reorganisation and dispersion was considered as present when a difference of 2 or more grades was observed between left and right side for more than half the levels and a gradient if similar differences in grades between any two levels in one hemisphere (for details of grades refer to main text). CB = calbindin, CR = calretinin, NPY = Neuropeptide Y, R = right side, L = left side, N = not present, Y = present; if present, the specific details of changes are provided.
