# Supplementary material for: Variability of sclerosis along the longitudinal hippocampal axis in epilepsy: A post mortem study
Source: Epilepsy Res. 2012 Nov;102(1-2):45–59. doi: 10.1016/j.eplepsyres.2012.04.015 (PMC3500681; doi:10.1016/j.eplepsyres.2012.04.015)
Supplement: Figure 1 (Supplemental file) — Dentate gyrus reorganisation in HS cases observed with calretinin, calbindin and Neuropeptide Y (NPY) and the pattern of granule cell dispersion (GCD) along the hippocampal axis. Each is depicted in a different colour which is shown in four shades and corresponds to the grade of alterations. Calretinin (green): Grade 0 = normal pattern (palest shade), Grade 1 = reduction of infragranular fibres/increase in supragranular fibres and reduction in infragranular fibres (mid shade), Grade 2 = supragranular sprouts in the inner molecular layer (IML) (darker shade), Grade 3 = extensive fibres in IML and outer molecular layer (OML) (darkest shade). Calbindin (Orange): Grade 0 = normal pattern (palest shade), Grade 1 = calbindin mainly lost in granule cells (mid shade), Grade 2 = Basal granule cells calbindin negative, distal cells calbindin positive (darkest shade). NPY (Purple): Grade 0 = normal (palest shade), Grade 1 = some increase in fibres in IML with maintenance of difference between IML/OML (mid shade), Grade 2 = marked sprouts in IML and OML (darker shade), Grade 3 = sprouts in OML and infragranular region (darkest shade). GCD (blue) is graded as predominant cell loss (lightest shade), normal = Grade 0 (mid shade), Grade 1 = mild dispersion (darker shade) and Grade 2 = severe dispersion (darkest shade). The changes are shown in the left hippocampus (top row) and right hippocampus (bottom row) along the anterior to posterior longitudinal axis of the hippocampus from levels 4 to 10 (see text for details of levels). [file mmc1.doc]

| Group | | Symmetrical / A-P grad | | | | | | | | | | | | | |  | Symmetrical /No grad | | | | | | | | | | | | | |  | Asymmetrical / AP grad | | | | | | | | |  | Asymmetrical /No Grad | | | | | | | | |
| --- | --- | --- | --- | --- | --- | --- | --- | --- | --- | --- | --- | --- | --- | --- | --- | --- | --- | --- | --- | --- | --- | --- | --- | --- | --- | --- | --- | --- | --- | --- | --- | --- | --- | --- | --- | --- | --- | --- | --- | --- | --- | --- | --- | --- | --- | --- | --- | --- | --- | --- |
| Case | | EP108 | | | |  | EP054 | | | |  | EP200 | | | |  | EP082 | | | |  | EP038 | | | |  | EP254 | | | |  | EP055 | | | |  | EP019 | | | |  | EP016 | | | |  | EP002 | | | |
| Level A-P¯ | | CR | CB | NPY | GCD |  | CR | CB | NPY | GCD |  | CR | CB | NPY | GCD |  | CR | CB | NPY | GCD |  | CR | CB | NPY | GCD |  | CR | CB | NPY | GCD |  | CR | CB | NPY | GCD |  | CR | CB | NPY | GCD |  | CR | CB | NPY | GCD |  | CR | CB | NPY | GCD |
| Left Dentate gyrus | 4 |  |  |  |  |  |  |  |  |  |  |  |  |  |  |  |  |  |  |  |  |  |  |  |  |  |  |  |  |  |  |  |  |  |  |  |  |  |  |  |  |  |  |  |  |  |  |  |  |  |
|  | 5 |  |  |  |  |  |  |  |  |  |  |  |  |  |  |  |  |  |  |  |  |  |  |  |  |  |  |  |  |  |  |  |  |  |  |  |  |  |  |  |  |  |  |  |  |  |  |  |  |  |
|  | 6 |  |  |  |  |  |  |  |  |  |  |  |  |  |  |  |  |  |  |  |  |  |  |  |  |  |  |  |  |  |  |  |  |  |  |  |  |  |  |  |  |  |  |  |  |  |  |  |  |  |
|  | 7 |  |  |  |  |  |  |  |  |  |  |  |  |  |  |  |  |  |  |  |  |  |  |  |  |  |  |  |  |  |  |  |  |  |  |  |  |  |  |  |  |  |  |  |  |  |  |  |  |  |
|  | 8 |  |  |  |  |  |  |  |  |  |  |  |  |  |  |  |  |  |  |  |  |  |  |  |  |  |  |  |  |  |  |  |  |  |  |  |  |  |  |  |  |  |  |  |  |  |  |  |  |  |
|  | 9 |  |  |  |  |  |  |  |  |  |  |  |  |  |  |  |  |  |  |  |  |  |  |  |  |  |  |  |  |  |  |  |  |  |  |  |  |  |  |  |  |  |  |  |  |  |  |  |  |  |
|  | 10 |  |  |  |  |  |  |  |  |  |  |  |  |  |  |  |  |  |  |  |  |  |  |  |  |  |  |  |  |  |  |  |  |  |  |  |  |  |  |  |  |  |  |  |  |  |  |  |  |  |
|  |  |  |  |  |  |  |  |  |  |  |  |  |  |  |  |  |  |  |  |  |  |  |  |  |  |  |  |  |  |  |  |  |  |  |  |  |  |  |  |  |  |  |  |  |  |  |  |  |  |  |
| Right Dentate gyrus | 4 |  |  |  |  |  |  |  |  |  |  |  |  |  |  |  |  |  |  |  |  |  |  |  |  |  |  |  |  |  |  |  |  |  |  |  |  |  |  |  |  |  |  |  |  |  |  |  |  |  |
|  | 5 |  |  |  |  |  |  |  |  |  |  |  |  |  |  |  |  |  |  |  |  |  |  |  |  |  |  |  |  |  |  |  |  |  |  |  |  |  |  |  |  |  |  |  |  |  |  |  |  |  |
|  | 6 |  |  |  |  |  |  |  |  |  |  |  |  |  |  |  |  |  |  |  |  |  |  |  |  |  |  |  |  |  |  |  |  |  |  |  |  |  |  |  |  |  |  |  |  |  |  |  |  |  |
|  | 7 |  |  |  |  |  |  |  |  |  |  |  |  |  |  |  |  |  |  |  |  |  |  |  |  |  |  |  |  |  |  |  |  |  |  |  |  |  |  |  |  |  |  |  |  |  |  |  |  |  |
|  | 8 |  |  |  |  |  |  |  |  |  |  |  |  |  |  |  |  |  |  |  |  |  |  |  |  |  |  |  |  |  |  |  |  |  |  |  |  |  |  |  |  |  |  |  |  |  |  |  |  |  |
|  | 9 |  |  |  |  |  |  |  |  |  |  |  |  |  |  |  |  |  |  |  |  |  |  |  |  |  |  |  |  |  |  |  |  |  |  |  |  |  |  |  |  |  |  |  |  |  |  |  |  |  |
|  | 10 |  |  |  |  |  |  |  |  |  |  |  |  |  |  |  |  |  |  |  |  |  |  |  |  |  |  |  |  |  |  |  |  |  |  |  |  |  |  |  |  |  |  |  |  |  |  |  |  |  |

Figure 1
