# Supplementary material for: Variability of sclerosis along the longitudinal hippocampal axis in epilepsy: A post mortem study
Source: Epilepsy Res. 2012 Nov;102(1-2):45–59. doi: 10.1016/j.eplepsyres.2012.04.015 (PMC3500681; doi:10.1016/j.eplepsyres.2012.04.015)
Supplement: Table 1 (Supplementary table) — Mean control values for neuronal densities at different levels for the left and right hippocampus from three controls (six hippocampal specimens) (*NPY stained sections not available for these levels but from next anterior level). [file mmc2.doc]

| **Hippocampal Levels** | **CRESYL VIOLET**  **CV**  **x10^-5^/ µm^3^** | | **CALRETININ**  **CR**  **x10^-5^/ µm^2^** | | **CALBINDIN**  **CB**  **x10^-5^/ µm^2^** | | **NEUROPEPTIDE Y**  **NPY**  **x10^-5^/ µm^2^** |
| --- | --- | --- | --- | --- | --- | --- | --- |
|  | **CA1** | **CA4** | **CA1** | **CA4** | **CA1** | **CA4** | **CA4** |
| 4 | 0.63 | 0.28 | 2.01 | .99 | 1.7 | 0.55 | 3.75 |
| 5 | 1.3 | 0.49 | .54 | .42 | 0.79 | 0.58 | 0.78 |
| 6 | .76 | 0.70 | 2.36 | 1.44 | 1.18 | 0.62 | *0.78 |
| 7 | 1.14 | 0.43 | 1.74 | 1.43 | 0.82 | 0.71 | 1.1 |
| 8 | 2.63 | 0.61 | 1.87 | 1.42 | 1.43 | 0.65 | 0.76 |
| 9 | 1.12 | 1.9 | 1.93 | 1.75 | 0.78 | 0.67 | 1.33 |
| 10 | 1.10 | 0.76 | 2.63 | 2.54 | 1.22 | 0.32 | *1.33 |

Table 1 (Supplementary table). Mean control values for neuronal densities at different levels for the left and right hippocampus from three controls (six hippocampal specimens). (* NPY stained sections not available for these levels but from next anterior level).
