# Supplementary material for: Variability of sclerosis along the longitudinal hippocampal axis in epilepsy: A post mortem study
Source: Epilepsy Res. 2012 Nov;102(1-2):45–59. doi: 10.1016/j.eplepsyres.2012.04.015 (PMC3500681; doi:10.1016/j.eplepsyres.2012.04.015)
Supplement: Table 2 (Supplementary table) — Four groups of hippocampal sclerosis (HS) based on the predominant pattern of left: right asymmetry and the gradient of neuronal and interneuronal loss along the longitudinal axis. Asymmetry was defined as a difference of 2 grades** between left and right sides for more than 50% of levels examined. An A-P gradient was defined as a difference of 2 grades** or more between any levels from one hemisphere. [A = cell loss predominant in anterior levels (levels 4–6), P = cell loss predominant in posterior levels (9, 10) and M = cell loss predominant in mid levels (7, 8)]. *Limited levels from this case as post. temporal lobectomy. **The four grades were 0–25%, 25–50%, 50–75% or 75%+ of neuronal loss relative to control values for that level. Asymmetry for dentate gyrus reorganisation and dispersion was considered as present when a difference of 2 or more grades was observed between left and right side for more than half the levels and a gradient if similar differences in grades between any two levels in one hemisphere (for details of grades refer to main text). CB = calbindin, CR = calretinin, NPY = Neuropeptide Y, R = right side, L = left side, N = not present, Y = present; if present, the specific details of changes are provided. [file mmc3.doc]

| **Case** | **NEURONAL DENSITIES** | | | | | | | | | | | | **DENTATE GYRUS REORGANISATION** | | | | | | | | **Predominant pattern** | **Group** |
| --- | --- | --- | --- | --- | --- | --- | --- | --- | --- | --- | --- | --- | --- | --- | --- | --- | --- | --- | --- | --- | --- | --- |
|  | **ASYMMETRY LEFT : RIGHT** | | | | **ANTERIOR : POSTERIOR GRADIENT** | | | | | | | | **ASYMMETRY LEFT : RIGHT** | | | | **ANTERIOR : POSTERIOR GRADIENT** | | | |  |  |
|  | **Pyramidal CA1** | **Pyramidal CA4** | **Interneurones CA1** | **Interneurones CA4** | **Pyramidal (CV) CA1 Left** | **Pyramidal (CV) CA1 Right** | **Pyramidal (CV) CA4 Left** | **Pyramidal (CV) CA4 Right** | **Interneurones CA1 Left** | **Interneurones CA1 Right** | **Interneurones CA4 Left** | **Interneurones CA4 Right** | **Calretinin** | **Calbindin** | **Neuropeptide Y** | **Granule cell disp.** | **Calretinin** | **Calbindin** | **Neuropeptide Y** | **Granule cell disp.** |  |  |
| EP108 | N | N | N | N | A>P | A>P | A>P | A>P | N | N | CR A>P | CR A>P | N | N | N | N | A>P  (L&R) | A>P  (R) | M  (R) | A>P  (R) | **Symmetrical**  **Gradient A>P** | **1** |
| EP054 | N | N | N | N | A>P | M | M | A | CB  A>P | CB  A>P | CB  A>P | CB  P>A | N | N | Y | N | A>P  (L&R) | A>P  (L&R) | A>P  (R)  M  (L) | A.P  (R) | **Symmetrical**  **Gradient A>P** |  |
| EP200 | N | N | CB  L>R | CB  L>R | M | M | P>A | N | CR, CB  A>P | CR  A>P | CR  A>P  NPY  P>A | CR,NPY  A>P | L>R | L>R | L>R | L>R | A>P  (L) | N | A>P  (L) | N | **Symmetrical (pyramidal)**  **Gradient A>P** |  |
| EP082 | N | R>L | N | N | M | A>P | N | N | CR  P | N | CR  P | CB  M | N | N | N | N | N | N | N | N | **Symmetrical**  **No gradient** | **2** |
| EP038 | R>L | N | N | N | P>A | N | N | N | N | N | N | NPY  P>A | Y | N | N | R>L | P>A  (L)  A>P  (R) | N | A>P  (R) | N | **Symmetrical**  **No gradient** |  |
| EP254 | N | N | N | - | N | N | N | - | CB  A>P | N | N | - | - | - | - | - | N | N | N | N | **Symmetrical**  **No gradient** |  |
| EP055 | L>R | L>R | N | N | N | M | A>P | P | CB  P>A | N | CR, NPY  M  CB  A>P | CB, NPY  M | L>R | L>R | N | N | A>P L | P>A  (L) | A>P  (L&R) | N | **Asymmetrical**  **Gradient** | **3** |
| EP019 | L>R | L>R | N | N | A>P | N | N | N | CR  P>A  CB  A>P | N | CB, CR, NPY  (P) | N | L>R | N | N | N | A>P  (L&R) | P>A  (L) | A>P  (L&R) | A>P  (L) | **Asymmetrical**  **Gradient** |  |
| EP016 | R>L | R>L | CB  L>R | N | N | N | N | N | CB  M | N | CB  M | CR  A>P  CB  P>A | R>L | N | R>L | N | N | A>P  (L&R) | A>P  (R) | N | **Asymmetrical**  **No gradient** | **4** |
| EP002 | R>L | R>L | N | NPY  R>L | N | A>P | P>A | N | CR  P>A  CB  M | N | N | CB  M | R>L | R>L | R>L | N | A>P  (R) | M  (R) | N | N | **Asymmetrical**  **No gradient** |  |

Table 2 (Supplementary table). Four groups of hippocampal sclerosis (HS) based on the predominant pattern of left: right asymmetry and the gradient of neuronal and interneuronal loss along the longitudinal axis. Asymmetry was defined as a difference of 2 grades** between left and right sides for more than 50% of levels examined. An A-P gradient was defined as a difference of 2 grades ** or more between any levels from one hemisphere. [A= cell loss predominant in anterior levels (levels 4,5,6), P = cell loss predominant in posterior levels (9,10) and M = cell loss predominant in mid levels. (7,8).] *Limited levels from this case as post. temporal lobectomy. **The four grades were 0-25%, 25-50%, 50-75% or 75%+ of neuronal loss relative to control values for that level. Asymmetry for dentate gyrus reorganisation and dispersion was considered as present when a difference of 2 or more grades was observed between left and right side for more than half the levels and a gradient if similar differences in grades between any two levels in one hemisphere (for details of grades refer to main text). CB=calbindin, CR=Calretinin, NPY=Neuropeptide Y, R=right side, L=left side, N=Not present Y=present; if present, the specific details of changes are provided.
